# Role of NLRP3 inflammasomes in monocyte and microglial recruitments in choroidal neovascularization

**DOI:** 10.21203/rs.3.rs-3318233/v1

**Published:** 2023-09-09

**Authors:** Blake W. Dieckmann, Marcell E. Paguaga, Gary W. McCollum, John S. Penn, Imam Uddin

**Affiliations:** Vanderbilt University School of Medicine; Vanderbilt University School of Medicine; Vanderbilt University School of Medicine; Vanderbilt University School of Medicine; Vanderbilt University Medical Center

**Keywords:** Age-related macular degeneration, monocyte, microglia, laser-induced choroidal neovascularization, macrophage, NLRP3 inflammasome, NLRP3 inhibitor, MCC950

## Abstract

Though the pathogenesis of choroidal neovascularization (CNV) is largely unknown in age-related macular degeneration (AMD), inflammasomes may contribute to CNV development and progression. To understand the role NLRP3 inflammasomes in CNV, we used *Ccr2*^*RFP*^*Cx3cr1*^*GFP*^ dual-reporter mice to characterize migration of *Ccr2*^*RFP*^ positive monocytes and *Cx3cr1*^*GFP*^ positive microglial cells into CNV lesions after laser-induced rupture of Bruch’s membrane. MCC950 was used as NLRP3 inhibitor. Immunostaining was used to confirm localization of NLRP3 inflammasomes in the LCNV lesions. Confocal microscopy was used to image and quantify LCNV volumes. ELISA and qRT-PCR were used to confirm the activation of NLRP3 by monitoring the expression of IL-1β protein and mRNA in choroidal tissues from LCNV mice. In addition, NLRP3 (−/−) LCNV mice were used to investigate whether NLRP3 inflammasomes contribute to the development of LCNV lesions. We observed that RFP positive monocyte-derived macrophages and GFP positive microglia-derived macrophages, in addition to other cell types, were localized in LCNV lesions at day 7 post-laser injury. In addition, NLRP3 inflammasomes are associated with LCNV lesions. Inhibition of NLRP3 inflammasomes, using MCC950, caused an increased Ccr2^RFP^ positive macrophages, Cx3cr1^GFP^ positive microglia, and other cells resulting in an increase in total lesion size. NLRP3 (−/−) LCNV mice, showed significantly increased lesion size compared to age-matched controls. Inhibition of NLRP3, resulted in decreased IL-1β mRNA and protein expression in the choroidal tissues, suggesting that increased lesion size may not be directly related to IL-1β.

## 1. Introduction

Age-related macular degeneration (AMD) is a vision threatening condition affecting older adults, often resulting in blindness, in developed countries.^1,2^ AMD is classified into two major forms: dry AMD and neovascular or “wet” AMD. Even though only 10% of AMD cases are “wet”, they contribute to about 90% of the vision loss in AMD. A key characteristic of “wet” AMD is choroidal neovascularization (CNV) in the macula causing severe vision loss.^3^ Though the pathogenesis of CNV is largely unknown, inflammasomes may contribute to its onset and progression. Currently, CNV is managed by intravitreal administration of anti-VEGF therapy. However, about 50% of “wet” AMD patients exhibit an incomplete response to anti-VEGF drugs. NOD-, LRR-, and PYD-containing protein 3 (NLRP3) inflammasome, a macromolecular complex that forms and induces inflammation in response to pathogens and cellular damage, is associated with CNV lesions.^4,5^ However, the role of NLRP3 is largely unknown. We hypothesized that NLRP3 inflammasomes contribute to CNV by regulating monocyte and microglial migration into the subretinal space.

**Figure 1:**
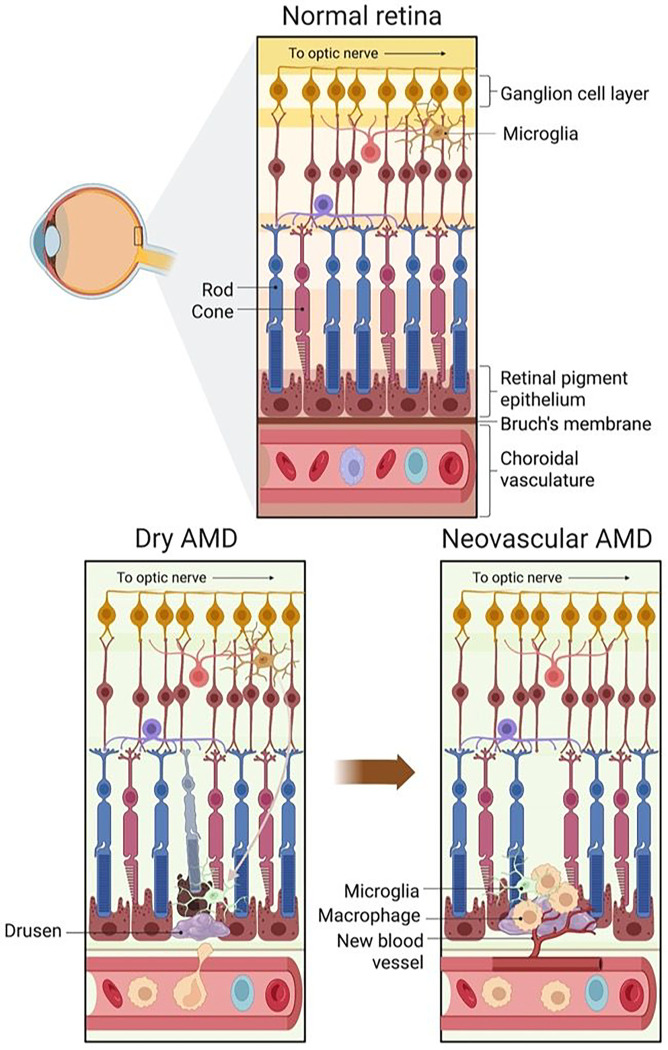
Changes in retinal structures in early-stage or dry AMD, and late-stage or neovascular AMD. In normal retina, microglia migrate into and out of the subretinal space. Dry AMD is associated with the accumulation of drusen in subretinal space, accumulation of microglia and macrophages and a thickened Bruch’s membrane. In neovascular AMD, Bruch’s membrane breakdown leads to choroidal neovascularization (CNV), macrophage accumulation and photoreceptor degeneration. Increased expression of NLRP3 inflammasomes is associated with AMD progression and could be an important target for molecular imaging of inflammation to predict the risk of CNV development and progression.

The NLRP3 inflammasomes consist of three components: 1) NLRP3, 2) apoptosis-associated speck containing a CARD (ASC), and 3) caspase-1. When NLRP3 inflammasome formation is triggered, it enzymatically activates pro-angiogenic cytokines such as interleukin-1β (IL-1β),^6^ which can promote other pro-angiogenic molecules such as VEGF. Figure 1 highlights the key features of AMD and how macrophages may contribute to the progression of CNV by a mechanism regulated by NLRP3 inflammasomes. Activated NLRP3 inflammasomes are associated with macrophages that infiltrate into the CNV lesions in *VEGF-A*^*hyper*^ mice, a genetic mouse model of AMD.^7^ Genetic inactivation of NLRP3 in these VEGF-A^*hyper*^ mice reduced the number of CNV lesions.^8^ However, role of NLRP3 inflammasomes in monocyte and microglial recruitment is not characterized. One major challenge to study the role of monocytes/microglia in AMD, relates to the difficulties associated with distinguishing between peripheral monocyte derived macrophages and resident microglia.^9^ In addition, both of these two cell types present common cell markers including F4/80.^7^ In the current study, we have used *Ccr2*^*RFP*^*Cx3cr1*^*GFP*^ dual-reporter mice, where resident microglia are tagged with green fluorescent protein (GFP) and circulating monocytes are tagged with red fluorescent protein (RFP). Therefore, we can distinguish between these two cell types while investigating the role of NLRP3.^10^ To study the pathogenesis of AMD, several animal models have been reported in the literature.^11^ A reliable and reproducible model is the laser-induced choroidal neovascularization (LCNV).^12^ Despite missing key components of wet AMD, this model has been used in the development of therapies and the elucidation of molecular pathways in neovascularization.^13^ In this study, *Ccr2*^*RFP*^*Cx3cr1*^*GFP*^ dual-reporter mice were tested using the LCNV protocol, allowing us to differentiate between resident microglia and monocyte-derived macrophages that are recruited to the CNV lesions.^14^ In addition, MCC950 is tested as NLRP3 inhibitor in the LCNV model using *Ccr2*^*RFP*^*Cx3cr1*^*GFP*^ dual-reporter mice. MCC950 is an efficient NLRP3 inhibitor, which binds to NLRP3’s NACHT domain and blocks the formation of the inflammasome complex. This inhibitor has been shown to alleviate symptoms in murine models of inflammatory diseases.^15,16^ Notably, MCC950 mitigated retinal neovascularization in murine diabetic retinopathy,^17^ and murine oxygen-induced retinopathy^18^. In the present study, we examined the role of NLRP3 in monocyte recruitment and its effects on CNV developments.

## 2. Materials and Methods

### 2.1. Reagents and drug formulations

All reagents were purchased and used as received unless otherwise indicated. MCC950 sodium salt was acquired from Selleckchem.com (Houston, TX; Catalog No. S7809) and formulated to 4.4 mg/mL in a vehicle consisting of 10% dimethyl sulfoxide (DMSO, Sigma, Burlington, MA; Catalog No. D8418) and 90% Dulbecco’s phosphate buffered saline (DPBS, Gibco, Waltham, MA; Catalog No. 14190–144). Ketamine (NDC 0409–2051-15) was obtained from Hospira, Inc. (Lake Forest, IL). Xylazine (NDC 593990110–20), proparacaine hydrochloride (NDC 17478–263-12), and fluorescein (NDC 17478–253-10) were acquired from Akorn, Inc. (Lake Forest, IL). Tropicamide (NDC 61314–354-01) was purchased from Sandoz (Basel, Switzerland) and phenylephrine (NDC 42707–102-15) was obtained from Paragon BioTeck, Inc. (Portland, OR). GenTeal Severe gel (0065–8064-01) was purchased from Alcon laboratories, Inc (Fort Worth, Texas).

### 2.2. Animals

C57BL/6J and B6.129(Cg)-Cx3cr1^tm1Litt^ Ccr2^tm2.1Ifc^/Jern (Ccr2^RFP^Cx3cr1^GFP)^J mice were purchased from Jackson Laboratories (Bar Harbor, MA). At the time of LCNV induction, C57BL/6 mice and C57BL/6-J GFP/RFP mice were 8–10 weeks old. Mice were group-housed according to their randomly assigned experimental treatment groups in ventilated cages maintained under a 12 h:12 h light:dark cycle at 22 ± 2°C within an institutional animal care facility. They were provided clean water (Nashville Metro Water Services, Nashville, TN) and a standard diet consisting of 4.5% fat (PicoLab Rodent Diet 5L0D; LabDiet, St. Louis, MO) ad libitum. They were humanely sacrificed by CO_2_-induced asphyxiation followed by cervical dislocation.

### 2.3. Anesthesia, pupillary dilation, and corneal numbing

Anesthesia, pupillary dilation, and corneal numbing were performed prior to the following procedures: Laser-induced choroidal neovascularization (LCNV) and electroretinography (ERG). Average 22 g weight mice (n = 12 per group) were injected intraperitoneally with a 70 μL solution consisting of a 1:1:2 mixture of ketamine (85.7 mg/kg):xylazine (17.9 mg/kg):saline. After induction of anesthesia, mice pupils were dilated with a drop of 0.5% tropicamide and 2.5% phenylephrine. Their corneas were numbed with a drop of 0.5% proparacaine. Prior to the start of LCNV, eyes were covered with GenTeal gel to maintain hydration during laser-injury and imaging. For ERG measurements, eyes were hydrated using artificial tears.

### 2.4. Laser-induced choroidal neovascularization (LCNV)

Bruch’s membrane was ruptured with a laser using the Phoenix MICRON Image Guided Laser system and the Phoenix Micron IV Retinal Imaging system (Phoenix-Micron, Inc, Bend, OR). Mice were anesthetized and pupils were dilated as described above. Laser-induced Bruch’s membrane rupture was created in four quadrants in each eye about two optic disc lengths away from the optic nerve head. Hemorrhage or irregular lesions were excluded. Laser setting for power, laser time, diameter are 350 mW, 80 ms, and 50 um respectively. On day 7 post-laser, mice were sacrificed, eyes were harvested, fixed overnight at 4 °C and dissected for immunostaining.

### 2.5. NLRP3 inhibition using MCC950

After induction of LCNV on day 0, mice received intraperitoneal injections of MCC950 (40 mg/kg) or vehicle for six days, starting 1 day post-laser injury. Mice were sacrificed on day 7 for ex vivo analysis.

### 2.7. Electroretinography (ERG)

On day 7 post-MCC950 injection, mice were dark-adapted overnight and assessed for retinal cell health using electroretinography (ERG). ERG measurements were performed according to our previously published methods.^5^ Briefly, after anesthesia, mouse eyes were dilated as described above. Mice were place on a warm platform within the Ganzfeld dome of a Diagnosys LLC Espion Electrophysiology system (Lowell, MA, USA). Mice were then exposed to flashes of light ranging from - 4 to 2 log cd.s/m2 and the amplitudes of a-wave and b-wave were measured from baseline to peak. The amplitude of the a-wave and b-wave were plotted as a function of luminance.

### 2.8. Molecular biology assays

RNA extraction was performed using RNAeasy Mini Kit according to manufacturer’s protocol (Qiagen, Hilden, Germany, Catalog No. 74106). cDNA synthesis was performed using High Capacity cDNA Reverse Transcription Kit (Thermo Fisher Scientific, Waltham, MA, Catalog No. 4368814) according to manufacturer’s protocol, using a MiniAMP Thermal Cycler (Thermo Fisher Scientific, Waltham, MA, Catalog No. A37834). Cycling parameters are as follows: 50 ul sample, 105°C cover, 25°C for 10 minutes, 37°C for 2 hrs, 85°C for 5 minutes, 4°C until sample collection. Quantitative reverse transcription polymerase chain reaction (qRT-PCR) was performed using QuantStudio 3 (Thermo Fisher Scientific, Waltham, MA, Catalog No. A28567) using the following primers: Beta-actin control (Life technologies, Carlsbad, CA, Catalog No. 4351315), IL-1β (Thermo Fisher Scientific, Waltham, MA, Catalog No. Mm00434228), and Vegfa (Thermo Fisher Scientific, Waltham, MA, Catalog No. Mm01281447). Cycling parameters are as follows: Initial temperature to 95°C in 4.1 °C/s, 20 seconds at 95°C, 1 second at 95°C, decrease of 3.17°C/s until 60°C, 20 seconds at 60°C, increase 4.14°C/s until 95°C, repeats 1 second and 20 second intervals at 95°C and 60°C respectively. The Mouse IL-1β Elisa Kit (Invitrogen, Waltham, MA, Catalog No. MBS6002) was used to quantify IL-1β in the tissue samples. Manufacturer’s protocol was followed. Eyes were enucleated, fixed in 10% neutral buffered formalin for 10 min, dissected (lens, cornea, iris removed, retina, choroid, and sclera remained), and homogenized in Pierce^™^ RIPA buffer (Thermo Scientific, Waltham, MA, Catalog No.8900) using disposable pestles (Bel-Art, Wayne, NJ, Catalog No. F199230001). Halt-protease Inhibitor Single-Use Cocktail 100x (Thermo Scientific, Waltham, MA, Catalog No. 78430) was used as protease Inhibitor.

### 2.9 Ex vivo imaging and 3-D reconstruction of LCNV lesion

On day 7 post-laser injury, a total of 24 eyes per group, were harvested and fixed in 10% formalin at 4° C overnight, and then RPE-choroid-sclera complex tissues were dissected. Tissues were blocked for 2–3 hours in blocking solution (10% donkey serum, 1 *%* BSA, 0.002% fish collagen in wash buffer solution). Tissue from NLRP3 (−/−) mice were stained with Dylight 647 conjugated Isolectin B4 (Catalog No. DL-1208-.5, Vector laboratories) at dilution 1:100. Tissue from one set Ccr2^RFP^Cx3cr1^GFP^ mice were stained with ICAM-2 primary antibody (Biolegend, Catalog No. 105602) overnight at 4°C with 1:100 dilution and followed by a 90 minute incubation with Alexa Fluor 647 anti-rat IgG (Catalog No. A48272, Life Technologies) in a 1:100 dilution. Another set of Ccr2^RFP^Cx3cr1^GFP^ mice were stained with anti-NLRP3 (Invitrogen, MA5–32255) overnight at 4°C at 1:70 and followed by a 90 minute incubation with Alexa Fluor 647 Anti-rabbit IgG, (Catalog No. A31573, Life technologies) in a 1:100 dilution. All tissue was washed after both primary and secondary antibody incubations.Tissues were washed 3 times for 5 minutes with wash buffer. Next tissue were flat-mounted on slides using Prolong Diamond Antifade mounting media with DAPI (Catalog No. P36962, Invitrogen), and imaged using confocal microscopy. All confocal images were taken on a Zeiss LSM 710 AxioObserver microscope (Carl Zeiss AG, Oberkochen, Germany) using EC Plan-Neofluar 20x/o.50 M27 objective. For the Ccr2^RFP^Cx3cr1^GFP^ tissues, images were take using same parameters for all tissues. For the GFP channel parameters are as follows: 488 nm laser at 2% power, pinhole 2.73 AU, detector gain 700. For the RFP channel: 561 nm laser at 10% power, detector gain 750. For the 649 channel: 633 nm laser at 2% power, detector gain 700.

3-D reconstruction of LCNV lesions was performed using IMARIS 10.0 (Oxford Instruments, Abingdon, UK). In brief, images were converted from CZI files to Imaris files using IMARIS image converter. Meta data was checked to make sure the images were converted correctly. For each channel (GFP RFP ICAM2–647) the surface creation tool was used for 3d reconstruction. Surface creation (3-D rendering) was performed automatically for all images and then each of the surfaces were examined to remove any background noise. The total number of voxels (pixels) of each 3D-rendered surface was given by the software. This was converted to um^3^ by multiplying the total number of voxels by voxel volume (1.0 um × 1.24 um × 1.24 um). A total of 46–58 lesions from 24 eyes per groups were used for the final data analysis. LCNV lesions with hemorrhage or irregular shapes were excluded from this analysis.

### 2.10. Statistical analysis

Graphpad Prism 9 was used to make all graphs and perform statistical analysis. Multiple comparison t-tests were performed using the two-stage Benamini, Krieger and Yekutieli method with Q = 1 %. Data were expressed as the mean ± SEM.

## 3. Results

### NLRP3 is expressed in LCNV lesions.

**Figure 2:**
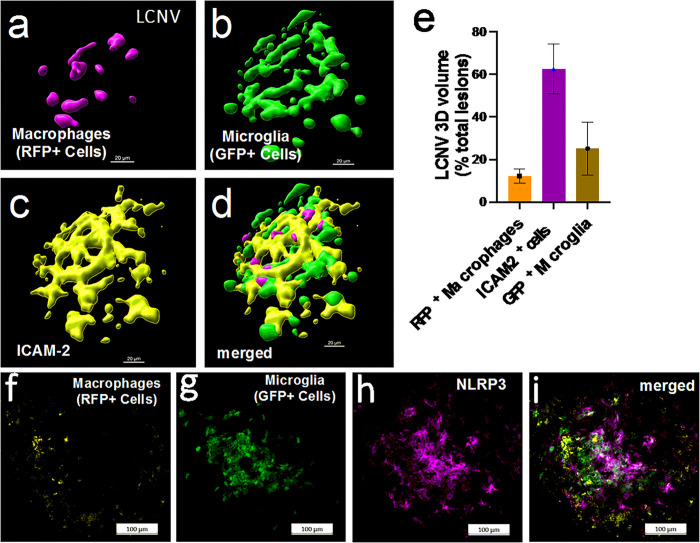
Migration of macrophages and microglia and expression of NLRP3 inflammasomes in LCNV lesions. We used Ccr2RFPCx3cr1 GFP dual-reporter mice to characterize migration of Ccr2RFP positive macrophages and Cx3cr1GFP positive microglia in LCNV at day-4 post laser injury. (a-e) Each LCNV lesion contains macrophages, about 15% of the total LCNV volume; activated microglia, about 20%; and ICAM-2 positive cells, about 65% of the total LCNV lesion volume. (f-i) The immunofluorescence imaging shows that NLRP3 inflammasomes are associated with LCNV lesions at day-4 post laser injury. These are representative images of 10 LCNV eyes.

As shown in Fig. 1, key features of wet AMD include migration of monocyte-derived macrophages and microglial cells into the subretinal space. We observed that in *Ccr2*^*RFP*^*Cx3cr1*^*GFP*^ dual-reporter mice and in WT C57BL/6J mice, the LCNV lesions were primarily composed of Isolectin B4 positive cells (mostly endothelial cells), GFP + microglia, and RFP + monocyte-derived macrophages (Fig. 2). In addition, NLRP3 expression is associated with LCNV lesions (Fig. 2f–i).

### NLRP3 inhibition increases choroidal neovascularization in LCNV animals.

**Figure 3:**
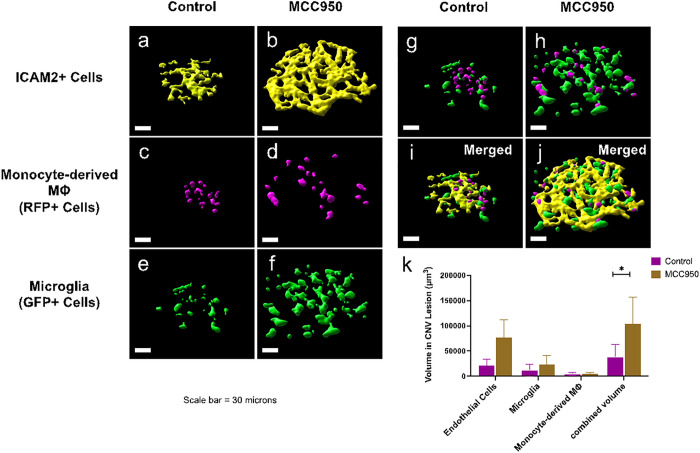
Role of NLRP3 inflammasomes in monocyte and microglial migration. (a-j) Representative Imaris reconstructed confocal images of the CNV lesions from control group and MCC950-treated group. (k) Inhibition of NLRP3 inflammasomes using MCC950 leads to increase in total volume of ICAM-2+ cells, Ccr2^RFP^ positive macrophages and Cx3cr1^GFP^ positive microglia, resulting in an increased lesion size. Volume of the specific cells in LCNV lesions were analyzed from Imaris generated 3D reconstruction of the corresponding confocal images. These are representative images of 10 LCNV eyes.

**Figure 4:**
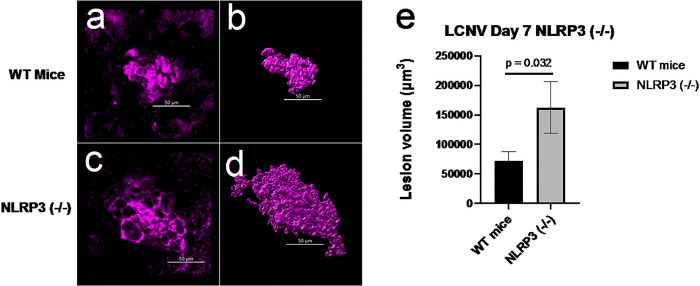
Role of NLRP3 inflammasomes in monocyte migration in NLRP3 (−/−) LCNV mice. (a-b) Representative confocal images of LCNV lesions in WT and NLRP3 (−/−) respectively. (c-d) Corresponding Imaris generated 3D-reconrstruction of the lesions (n = 19 and n = 13 for WT and NLRP3 (−/−) mice respectively. (e) The total volume of LCNV lesions were larger than the wild type age-matched controls.

**Figure 5:**
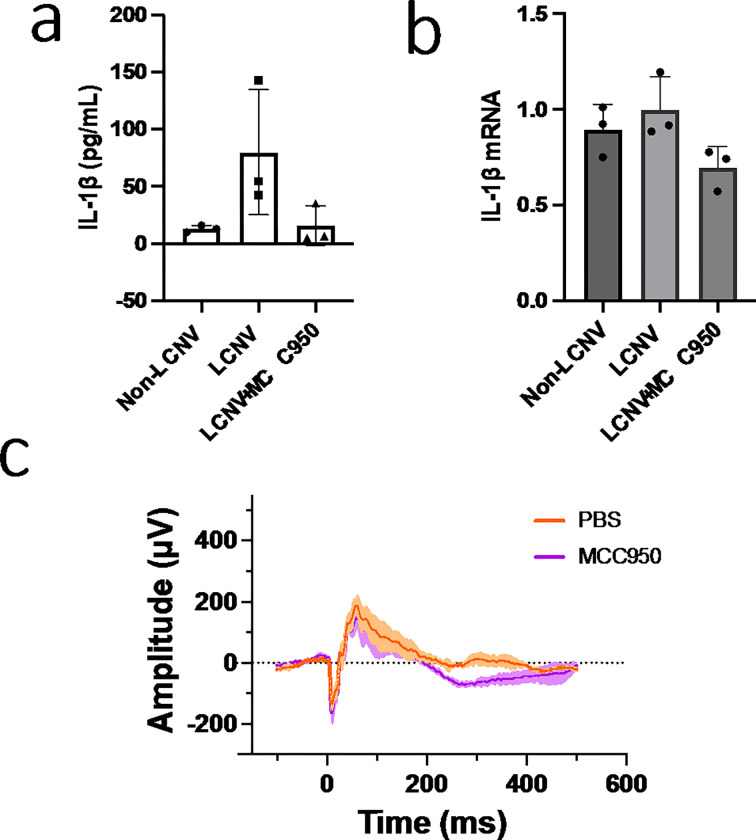
Expression of IL-1β in choroidal tissues isolated from LCNV animals at day-7 post-laser injury. (a, b) Both IL-1β protein and mRNA expressions were increased in LCNV tissues compared to non-lasered controls. Both IL-1β protein and mRNA expressions were decreased in MCC950 treated LCNV tissues, even though the volume of the CNV lesions were increased as shown in Figure 3. (c) In addition, we didn’t observe any changes in a-wave and b-wave amplitudes in ERG after MCC950 treatments, suggesting minimal effect on retinal cells.

NLRP3 inhibition caused an increased in volumes of CNV lesions in LCNV animals at day 7 post-laser injury (Fig. 3). *Ccr2*^*RFP*^*Cx3cr1*^*GFP*^ dual-reporter mice were either given MCC950 (NLRP3 inhibitor) or vehicle control for six days, from day 1 to day 6 after laser-induced damage to the Bruch’s membrane. On day 7 choroidal tissues were harvested, stained for IB4, mounted on slides, and imaged using confocal microscopy. We observed that LCNV lesions are primarily composed of Isolectin B4 positive cells (mostly endothelial cells), GFP + microglial cells, and RFP + monocyte-derived macrophages in *Ccr2*^*RFP*^*Cx3cr1*^*GFP*^ dual-reporter mice. There was a significant increase in volume of all these cells after NLRP3 inhibition, contributing to overall increased in CNV lesion size (Fig. 3). To further confirm the role of NLRP3 inflammasomes in LCNV lesion size increase, NLRP3 (−/−) mice were utilized. We observed that size of the LCNV lesions were significantly larger in NLRP3 (−/−) mice compared to WT mice, suggesting that NLRP3 inflammasomes may have contributions to CNV lesions (Fig. 4). These observations are in agreement with previously reported results.^19^ Interestingly, MCC950-treated mice showed decreased levels of IL1 b (Fig. 5a–b), a known pro-angiogenic molecule.^20^ NLRP3 activation leads to IL-1p production and may lead to downstream changes in VEGF expression.^19^ These observations suggests that the increased lesion size may not directly related to IL-1β .

### NLRP3 Inhibitors has no effect on retinal cell functions.

After NLRP3 inhibition using MCC950, we observed no significant changes in a-wave and b-wave amplitudes in response to flashes of light intensities as a functional test to detect retinal cell health using electroretinographic measurement (ERG) at day 7 post-laser injury (Fig. 5c). These data suggest that MCC950 has minimal effects on activation of other cells in the retina.

## Discussion

Wet AMD is a condition that is characterized by the key feature of CNV and currently is treated with agents that target VEGF. Even though they have proven beneficial effects in some cases, there is a large cohort of AMD patients that are refractory, resulting in disease progression and eventual vision loss.^21^ Therefore, understanding important drivers of choroidal neovascularization, other than VEGF, is necessary if outcomes are to ever be improved. In this study we examine how NLRP3 inflammasomes, microglia and monocyte-derived macrophages contribute to CNV development.^7^

First, we wanted to confirm the importance of NLRP3 and its effect on CNV development. In Ccr2^RFP^Cx3cr1^GFP^ mice, NLRP3 is expressed within the LCNV lesion as shown in Fig. 2. Next NLRP3 (−/−) mice were tested in the LCNV model to investigate the effects of NLRP3 activation on lesion volume. Indeed, NLRP3 knockout mice had an increase in lesion volume Fig. 4. These data confirm a study from Doyle et al, where they also see an increase in lesion size in this same strain of mice.^19^ There is controversy though whether NLRP3 is protective against CNV. Our data, in addition to Doyle et al^19^ suggest that NLRP3 is protective. But in mouse model where VEGF-A^*hyper*^ mice are crossed with NLRP3 null mice, researchers have found that there was an increased number of lesions.^8^ At first glance these results seem conflicting but what these data actually suggest that NLRP3 might have different roles to play at different times in CNV development. It is possible that NLRP3 can promote formation of CNV at early stage, while at a different time, in later stage of wet AMD for example, it can serve as protective role by decreasing CNV lesion size. The idea that the role of NLRP3 in CNV lesion formation versus progression could be different, is further supported by the fact that there was no reported difference in lesion numbers in the VEGF^hyper^/NLRP3 ^(−/−)^ mice compared to VEGF^hyper^ mice.^7^ This should be investigated further as it could affect the viability of NLRP3 as a drug target if its role shifts from detrimental to protective depending on the AMD progression.

To investigate the specific role(s) of NLRP3 on monocyte/microglial functions in CNV development, the *Ccr2*^*RFP*^*Cx3cr1*^*GFP*^ dual-reporter mice were used. This mouse model allows resident microglia to be clearly distinguished from macrophages derived from circulating monocytes.^10^ Our data clearly showed that both microglia and monocyte-derived macrophages are recruited to lesions and are present at the peak CNV development at day 7 post-laser injury (Fig. 3). Initially, these lesions were also stained with ICAM-2, a marker of endothelial cells,^22^ to help visualize the lesion’s volume. In addition, IB4 was used stain endothelial cells and other cells including microglia and macrophages, to visualize the LCNV volume.^23,24^

The LCNV model in *Ccr2*^*RFP*^*Cx3cr1*^*GFP*^ dual-reporter mice does have several limitations. Though *Ccr2*^*RFP*^*Cx3cr1*^*GFP*^ dual-reporter mice have been used to study retinal degeneration,^10^ it is a double knock-out/knock-in mouse model, so both CCR2 and Cx3cr1 have been deleted. Therefore, recruitment of monocytes through CCL2 signaling is impaired (**Supplementary Fig. 1**).^14^ Cx3cr1 modulates microglia activation in various ways depending on the neurological conditions.^25^ Therefore, microglia in this model could behave differently. **Supplementary Fig. 2** shows changes in microglial morphology of the course of LCNV model. Despite these limitations, it can still help us to show how monocytes are regulated by NLRP3 during choroidal neovascularization.

MCC950 treated mice have an increase in lesion volume compared to control mice (Fig. 3), similar to NLRP3 (−/−) mice (Fig. 4). This increased lesion volume was due to significant increases in microglia, monocyte-derived macrophages, and ICAM2+ cells. This suggests that NLRP3 plays a protective role against growth of CNV lesions, potentially by decreasing recruitment of monocyte-derived macrophages and microglia. Previous studies support this hypothesis by showing that lesion size decreases when macrophages are depleted.^26,27^ Other studies have shown that changes in microglia can contribute to CNV lesion size as well.^28^

Finally, we looked at pro-angiogenic signaling molecules that are associated with NLRP3 and CNV lesions. An important pro-angiogenic signaling molecule downstream of NLRP3 activation is IL-1β .^29^ In MCC950 treated mice, there was lower expression of both mRNA and secreted IL-1β protein (Fig. 5a–b), supporting that NLRP3 was inhibited by MCC950 despite an increase in total volume of the CNV lesions. Thus, our data suggest that this increased lesion size may not directly related to IL-1β.

In conclusion, wet AMD is a disease that is characterized by choroidal neovascularization. The anti-VEGF treatment used to treat this condition, is not effective for many AMD patients.^30^ Therefore. a greater understanding of choroidal neovascularization is needed to develop new potential therapies. Both NLRP3 and leukocytes have been investigated in the past for their roles in CNV. In our study we show that NLRP3 regulates monocyte-derived macrophages and microglial recruitment in choroidal neovascularization. Therefore, understanding this relationship between NLRP3 and leukocyte recruitment could further lead to a clear understanding and eventually develop new therapies to protect against choroidal neovascularization.

## Abbreviations

AMD: age-related macular degeneration
CNV: choroidal neovascularization
LCNV: laser-induced choroidal neovascularization
NLRP3: NOD-, LRR-, and PYD-containing protein
GFP: green fluorescent protein
RFP: red fluorescent protein
